# Aryl hydrocarbon receptor activation by *Lactobacillus reuteri* tryptophan metabolism alleviates *Escherichia coli*-induced mastitis in mice

**DOI:** 10.1371/journal.ppat.1009774

**Published:** 2021-07-23

**Authors:** Caijun Zhao, Xiaoyu Hu, Lijuan Bao, Keyi Wu, Lianjun Feng, Min Qiu, Haoyang Hao, Yunhe Fu, Naisheng Zhang

**Affiliations:** Department of Clinical Veterinary Medicine, College of Veterinary Medicine, Jilin University, Changchun, China; Vanderbilt University Medical Center, UNITED STATES

## Abstract

The intestinal microbiota has been associated with the occurrence and development of mastitis, which is one of the most serious diseases of lactating women and female animals, but the underlying mechanism has not yet been elucidated. Aryl hydrocarbon receptor (AhR) activation by microbiota tryptophan metabolism-derived ligands is involved in maintaining host homeostasis and resisting diseases. We investigated whether AhR activation by microbiota-metabolic ligands could influence mastitis development in mice. In this study, we found that AhR activation using Ficz ameliorated mastitis symptoms, which were related to limiting NF-κB activation and enhancing barrier function. Impaired AhR activation by disturbing the intestinal microbiota initiated mastitis, and processed *Escherichia coli* (*E*. *coli*)-induced mastitis in mice. Supplementation with dietary tryptophan attenuated the mastitis, but attenuation was inhibited by the intestinal microbiota abrogation, while administering tryptophan metabolites including IAld and indole but not IPA, rescued the tryptophan effects in dysbiotic mice. Supplementation with a *Lactobacillus reuteri* (*L*. *reuteri*) strain with the capacity to produce AhR ligands also improved *E*. *coli-*induced mastitis in an AhR-dependent manner. These findings provide evidence for novel therapeutic strategies for treating mastitis, and support the role of metabolites derived from the intestinal microbiota in improving distal disease.

## Author summary

Mastitis, a common disease for female during lactation period that could cause a health risk for humans or huge economic losses for animals, is caused by pathogen invasion. However, little is known about how host factors affect mastitis’ progression and outcome. Our previous study found that disrupting the intestinal microbiota exacerbated pathogen-induced mastitis, but the underlying mechanism is unclear. Host cell surface receptors recognize intestinal microbiota-metabolized ligands to mediate downstream signal transduction is the primary manner for microbiota-host interactions. In this study, we determined microbiota-mediated aryl hydrocarbon receptor (AhR) activation alleviated *E*. *coli*-induced mastitis in mice. Insufficient AhR ligands production by disrupting the intestinal microbiota contributed to mastitis progression, but compensation host with dietary tryptophan, AhR ligands or probiotic *Lactobacillus reuteri* protected mice from *E*. *coli*-induced mastitis by rescuing AhR activation. Our founding provides direct evidence for a potential strategy for treating mastitis and as a basis for targeting microbiota-host interaction for diseases intervention.

## Introduction

The complex ecosystem of the mammalian intestine consists of a dense and diverse mutualistic microorganism known as the intestinal microbiota has been at the forefront of research in human and animal health [1,2]. The intestinal microbiota has essential effects on physiological host homeostasis via crosstalk between the microbiota and the host, including metabolic function, immune regulation and barrier maintenance [1]. Emerging evidence has indicated that disruption of the fragile balance within the intestinal microbiota, termed dysbiosis, is involved in tremendous inflammatory and metabolic diseases in both intestine proximal and distant organs [1,3]. A previous study showed that a mouse fecal microbiota transplantation (FMT) from cows with mastitis, which is one of the most severe diseases for human and animals [4,5], caused systemic inflammation and mastitis in germ-free (GF) mice [6]. Moreover, our recent study revealed that disrupting the intestinal microbiota by antibiotic treatment increased susceptibility to *Staphylococcus aureus*-induced mastitis in mice [7]. These results suggest that the intestinal microbiota plays a significant role in mastitis progression [6–8], however, the way in which the intestinal microbiota mediates mastitis development and the potential molecular mechanisms, remain elusive.

Metabolite production derived from both commensal communities and host sources contributes to crosstalk between the host and microbiota for homeostasis [9,10]. Disturbed metabolite production is involved in the pathology of many diseases [9–12]. Therefore, the identification of metabolite derived from either microbes or host to supplement the change in composition or the function of the intestinal microbiota in different diseases is necessary not only for understanding microbe-host interactions but also to seek potential therapeutic targets for diseases.

Metabolites that mediating host effects often require host receptors. Among the array of host receptors affecting host homeostasis by sensing microbiota metabolites is the aryl hydrocarbon receptor (AhR), which participates in many aspects of host physiology and is activated predominantly by ligands metabolized from tryptophan originating from dietary sources through intestinal mutualistic microorganisms [13–15]. AhR activation is implicated in the maintenance of innate and adaptive immunity, the regulation of inflammatory responses and the improvement of epithelial barrier functions [16,17], and thus significantly affects individual health and disease risk. For example, impairments in dietary tryptophan-derived AhR ligand production by intestinal dysbiosis aggravated inflammatory bowel disease (IBD) [18,19], metabolic syndrome [20] and experimental autoimmune encephalomyelitis (EAE) [21,22]. The underlying mechanisms include reducing the expression of interleukin (IL)-22 and GLP-1 production or regulating nuclear factor (NF)-κB activation. Administering a tryptophan-enriched diet [21–23] or AhR agonists such as 6-formylindolo[3,2-b]carbazole (Ficz) [19,20], alleviated disease scores, as well as the probiotic *Lactobacillus reuteri* (*L*. *reuteri*) has the capacity to produce AhR ligands [19,20]. Apart from metabolic diseases, AhR activation leads to alternative susceptibility to infection and inflammation. Specifically, *AHR-/-* mice had increased pro-inflammatory cytokines production upon lipopolysaccharide (LPS) treatment [24,25]. Following invasions by different pathogens, including *Citrobacter rodentium*, *Clostridium difficile* and *Candida albicans*, AhR deficient mice displayed higher susceptibility which was attributed to inefficient IL-22 production [18,26,27]. Moreover, supplementation with IL-22, Ficz, a tryptophan-enriched diet or *L*. *reuteri* also reduced pathogen infections [18,26,28]. These suggest that inefficient AhR activation by inadequate AhR ligand production is conducive to disease progression. In turn, administering AhR ligand, a tryptophan-supplemented diet or AhR ligand producer may rescue the effects of AhR deficiency and improve disease. However, whether impaired AhR activation by intestinal dysbiosis contributes to the immunopathology of mastitis remains to be explored.

Given that dysfunction in the intestinal microbiota leads to mastitis [6,7], and accounts for damaged AhR activation [15], as well as on the trail indicating a role for abnormal tryptophan metabolism in mastitis [29], we hypothesized that impairing the induction of AhR ligands by disrupting of the intestinal microbiota could contribute to mastitis development.

In this study, we revealed that AhR was involved in the pathogenesis of *E*. *coli*-induced mastitis and that treating with Ficz ameliorated *E*. *coli*-induced mastitis in association with inhibiting inflammation and repairing the barrier function by activating AhR. Individuals treated with a cocktail of antibiotics (ABX) displayed lower levels of AhR activation. Disturbing the intestinal microbiota using ABX was enough to induce mastitis in mice, and worsen mastitis caused by *E*. *coli* stimulation. Tryptophan administration alleviates *E*. *coli*-induced mastitis but is reversed by ABX, while mastitis scores are reduced by supplementation with indole-3-aldehyde (IAld) and indole, but indole-3-propionic acid (IPA) exhibits only minor effects in dysbiotic mice. In addition, administering the *L*. *reuteri* strain alleviated *E*. *coli-*induced mastitis in an AhR-dependent manner.

## Results

### AhR activation alleviates the immunopathology of *E*. *coli*-induced mastitis

**Fig 1:**
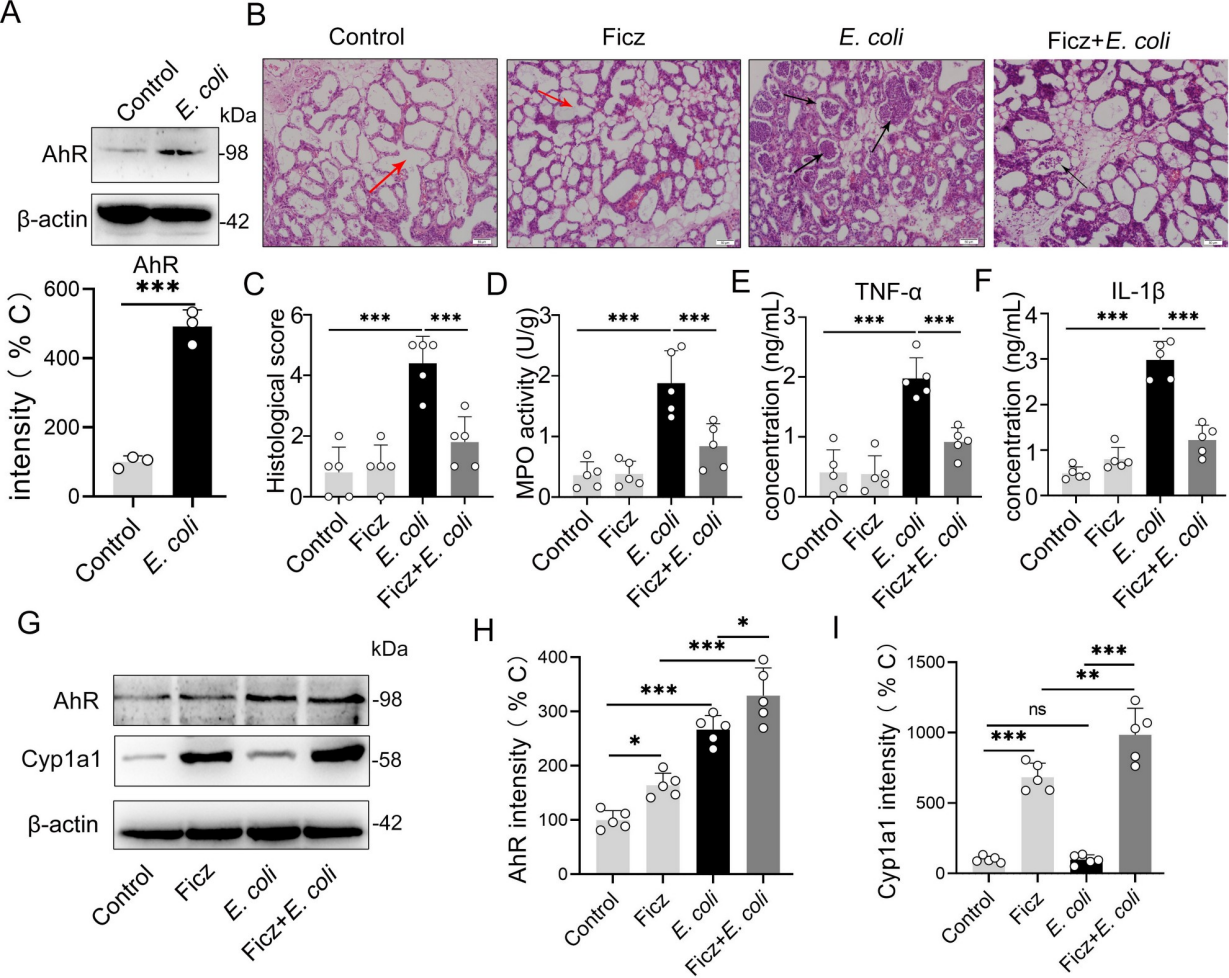
AhR activation by Ficz alleviates *E*. *coli*-induced mastitis. Mammary gland tissues were harvested from control, *E*. *coli* (10^7^ CFU/50 μL each breast duct) treated and Ficz (1 μg/mouse) pretreated mice 24 h after *E*. *coli* treatment. **(A**) AhR protein expression in mammary gland tissues from control and *E*. *coli-*treated mice was detected using western blotting (n = 3). **(B)** H&E-stained mammary gland tissue sections (scale bar, 50 μm). The red arrow indicates normal alveolar cells. The black arrow indicates the inflammatory cells infiltration. (**C)** Histological score according to the degree of alveolar injury and inflammatory cell infiltration (n = 5). (**D**) Levels of MPO activity (n = 5). The expression of pro-inflammatory cytokines TNF-α (**E**) and IL-1β (**F**) was measured by ELISA (n = 5). (**G-I**) AhR and Cyp1a1 levels in mammary gland tissues (n = 5). The results are shown as the means ± SEM. T test (**A**) and one-way analysis of variance (**C-F, H-I**) was used for statistical analysis. *p < 0.05, **p < 0.01 and ***p < 0.001 indicate significant differences from each group. ns, no significance.

To study the correlation between AhR activation and mastitis development, we first detected the AhR protein level in the mammary glands of mice with *E*. *coli*-induced mastitis. We found that mice treated with *E*. *coli* displayed higher AhR expression (**Fig 1A**), implying the regulatory role of the AhR pathway in *E*. *coli*-induced mastitis pathogenesis. To investigate the potential effects of the AhR pathway in regulating mastitis, we treated mice with Ficz, a classical agonist of AhR [19], followed by *E*. *coli* stimulation. We showed that *E*. *coli* treatment increased the mastitis inflammation score (**Fig 1B and 1C**), MPO activity (**Fig 1D**) and consistent expression of cytokines compared to the control group (**Fig 1E and 1F**), while Ficz pretreatment decreased the *E*. *coli*-induced inflammatory profiles compared to the *E*. *coli* treatment (**Fig 1B–1F**). We subsequently confirmed whether AhR activation was responsible for the protective effects of Ficz by detecting mammary gland AhR and Cyp1a1 protein levels, which is an AhR target gene that is responsible for AhR ligands depletion to prevent the overactive of AhR [16,17,30]. We found that Ficz increased the AhR and Cyp1a1 expression in the mammary glands (**Fig 1G–1I**). These results indicated that the activation of the AhR pathway by Ficz could limit the augmentation of mastitis caused by *E*. *coli*.

**Fig 2:**
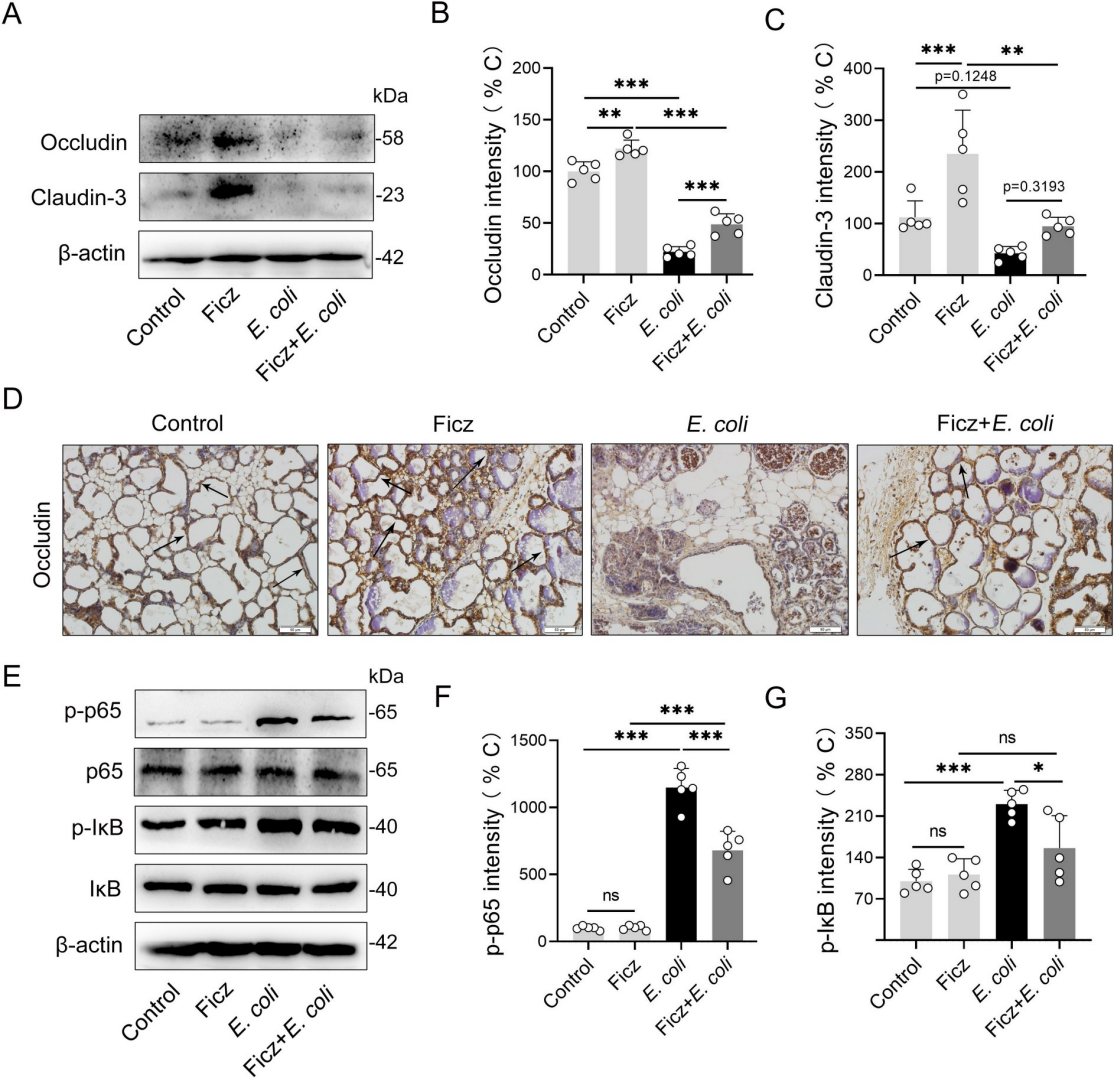
AhR activation by Ficz improves barrier integrity and limits inflammatory signaling in mice with *E*. *coli*-induced mastitis. Mice were treated with or without Ficz (1 μg/mouse) intraperitoneally 1 h before the *E*. *coli* was applied (10^7^ CFU/50 μL each breast duct). Twenty-four hours later, the mammary gland tissues were harvested and detected. (**A-C**) The levels of tight junction proteins, including occludin and claudin-3, in mammary glands were detected using western blots (n = 5). (**D**) Representative images of mammary gland immunohistochemistry (IHC) sections stained with occludin antibody (scale bar, 50 μm). The black arrow indicates the positive staining. **(E-G**) Protein levels of the NF-κB signaling pathway, including phosphorylated p65 and IκB, as well as p65 and IκB, which were measured by western blotting (n = 5). One-way analysis of variance was applied for statistical analysis of (**B-C)** and (**E-F)** and the date are presented as means ± SEM. *p < 0.05, **p < 0.01 and ***p < 0.001 mean significant differences from each group. ns, no significance.

AhR activation is associated with improving barrier integrity and limiting inflammatory signaling, which are involved in mastitis pathogenesis [7]. We further investigated the effects of AhR activation on barrier integrity and the NF-κB signaling pathway, which is the primary inflammatory signaling pathway involved in *E*. *coli*-induced mastitis development [31,32]. We showed that Ficz treated mice had higher levels of tight junction proteins, including occludin and claudin-3, than the control mice (**Fig 2A–2C**). Consistently, Ficz-treated mice rescued *E*. *coli*-induced barrier injury by increasing occludin and claudin-3 expression (**Fig 2A–2C**). We confirmed occludin level by immunochemistry and showed that Ficz increased the occludin expression with or without *E*. *coli* treatment (**Fig 2D**). Treating with *E*. *coli* increased the phosphorylation of p65 (p-p65) and IκB (p-IκB), indicating the activation of the NF-κB signaling pathway. However, Ficz treatment decreased the p-p65 and p-IκB expression induced by *E*. *coli* stimulation (**Fig 2E–2G**). To confirm the effects of AhR activation on the NF-κB pathway, we treated mouse mammary epithelial cells (MMECs) with Ficz. Consistently, we found that Ficz reduced p-p65 and p-IκB in a dose-dependent manner (S1A–S1C Fig), leading to AhR activation by ligands that inhibited the NF-κB pathway [33,34]. We also detected the effect of Ficz on *E*. *coli* growth and found no significance (S1D Fig). To verify the effects of AhR activation on *E*. *coli*-induced mastitis, we inhibited AhR activation by pretreating mice with CH223191, an AhR antagonist. We showed that CH223191 treated mice had higher inflammatory responses than *E*. *coli*-treated mice (S2A–S2E Fig). Similar studies have also demonstrated that treating with CH223191 exacerbated calcipotriol-induced dermatitis and colitis [19,35]. Likewise, the Ficz improvement of inflammatory features caused by *E*. *coli* was reversed by CH223191 pretreatment (S2A–S2E Fig), suggesting that inhibiting AhR signaling could worsen the inflammatory process. Similarly, CH223191 pretreatment exacerbated barrier injury and abolished the barrier protective effect of Ficz, which was characterized by reduced occludin and clasudin-3 expression (S2F–S2H Fig). Collectively, these results suggest that AhR activation alleviates *E*. *coli*-induced mastitis by improving tight junction protein expression and limiting NF-κB pathway activation.

### Impairing AhR activation by disturbing the intestinal microbiota is involved in mastitis occurrence and worsens *E*. *coli*-induced mastitis features

**Fig 3:**
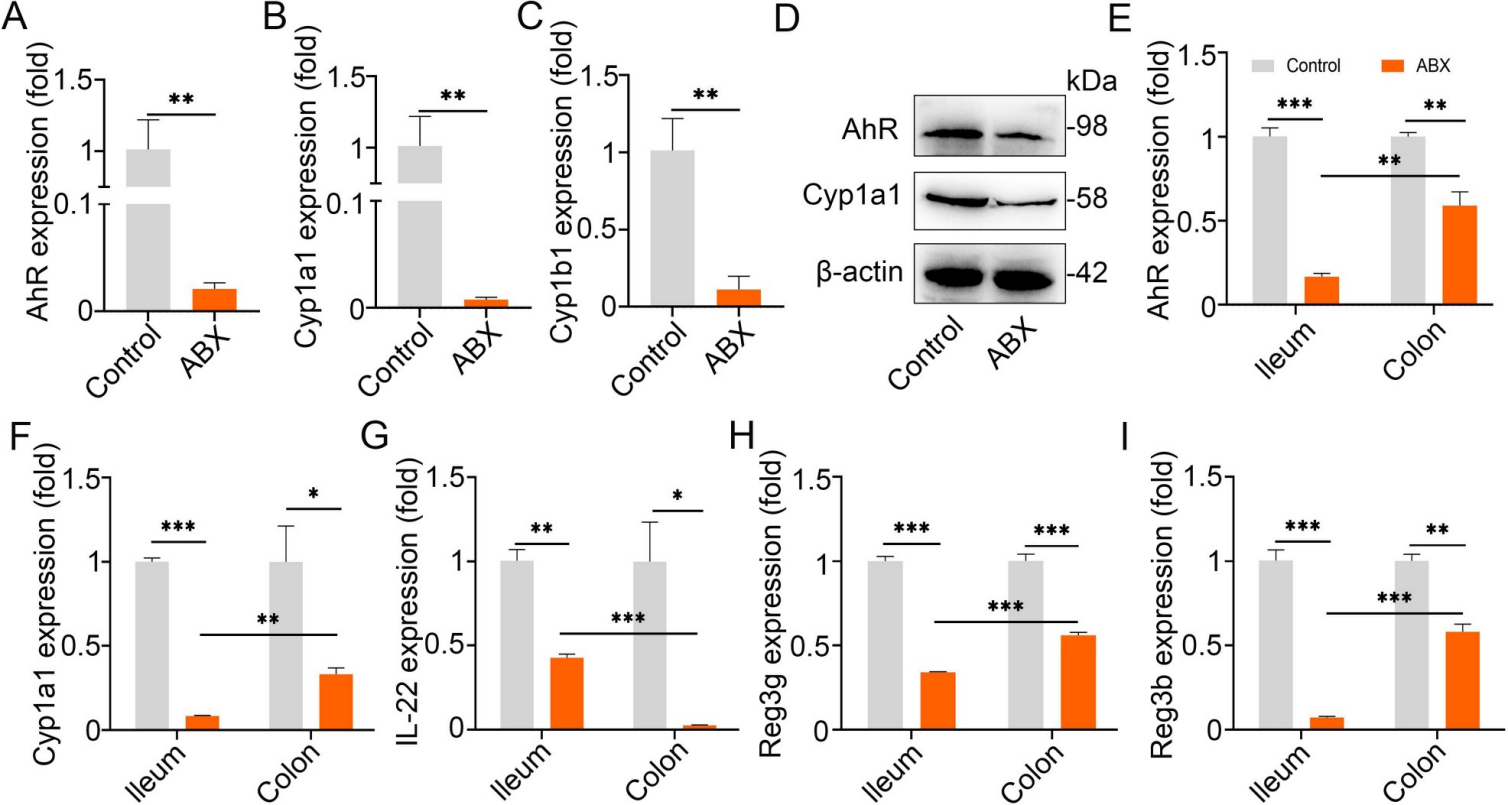
Gut dysbiosis impairs AhR activation. The mice were treated with a cocktail of antibiotics (ABX) containing 1 g/L ampicillin, neomycin sulfate and metronidazole and 0.5 g/L vancomycin for three weeks. Then, the mammary gland, ileum and colon tissues were collected. (**A-C**) The AhR, Cyp1a1 and Cyp1b1 mRNA levels in the mammary glands were identified using qPCR (n = 3). (**D)** Protein expression of AhR and Cyp1a1 in mammary glands of control and ABX-treated mice (n = 3). The levels of AhR (**E**), Cyp1a1 (**F**), IL-22 (**G**), Reg3g (**H**) and Reg3β (**I**) mRNA in ileum and colon tissues from control and ABX-treated mice were measured by qPCR (n = 3). Student’s t test were applied for statistical analysis in (**A-C)** and (**E-I)** and the date are shown as means ± SEM. *p < 0.05, **p < 0.01 and ***p < 0.001 indicate significant differences from each group. ABX, a cocktail of antibiotics.

To investigate the crosstalk between intestinal microbiota mediated AhR signaling and mastitis, we disrupted the intestinal microbiota by administering a cocktail of antibiotics (ABX) and then detected the AhR activation. We found that intestinally dysbiotic mice showed decreased AhR activation in their mammary gland at both mRNA and protein levels **(Fig 3A–3D)**. The AhR pathway can be activated by different factors, including microbiota-mediated ligands and inflammatory factors, such as lipopolysaccharide (LPS), leading to varied immune state [13,16,17]. To confirm the change in microbiota-mediated AhR activation, we detected the Cyp1a1 and Cyp1b1 expression in the mammary gland. We showed that gut dysbiotic mice had lower Cyp1a1 and Cyp1b1 expression than the control mice **(Fig 3B–3D)**, suggesting that the reduced AhR activation in the mammary gland of gut dysbiotic mice may respond to impaired intestinal microbiota-associated AhR ligand production, because Cyp1a1 activation is responsible for AhR ligand depletion [13,16,21,36]. To confirm our assumption, we detected the mRNA expression of AhR and Cyp1a1 in the ileum and colon tissues. Lower levels of AhR and Cyp1a1 mRNA were observed in gut dysbiotic mice than in control mice **(Fig 3E and 3F)**, and AhR mediated interleukin (IL)-22 and its target gene Reg3g and Reg3b levels **(Fig 3G–3I)**. These results suggest that gut dysbiotic mice experienced impaired AhR activation and intestinal barrier integrity, implying an increased systemic inflammation state.

**Fig 4:**
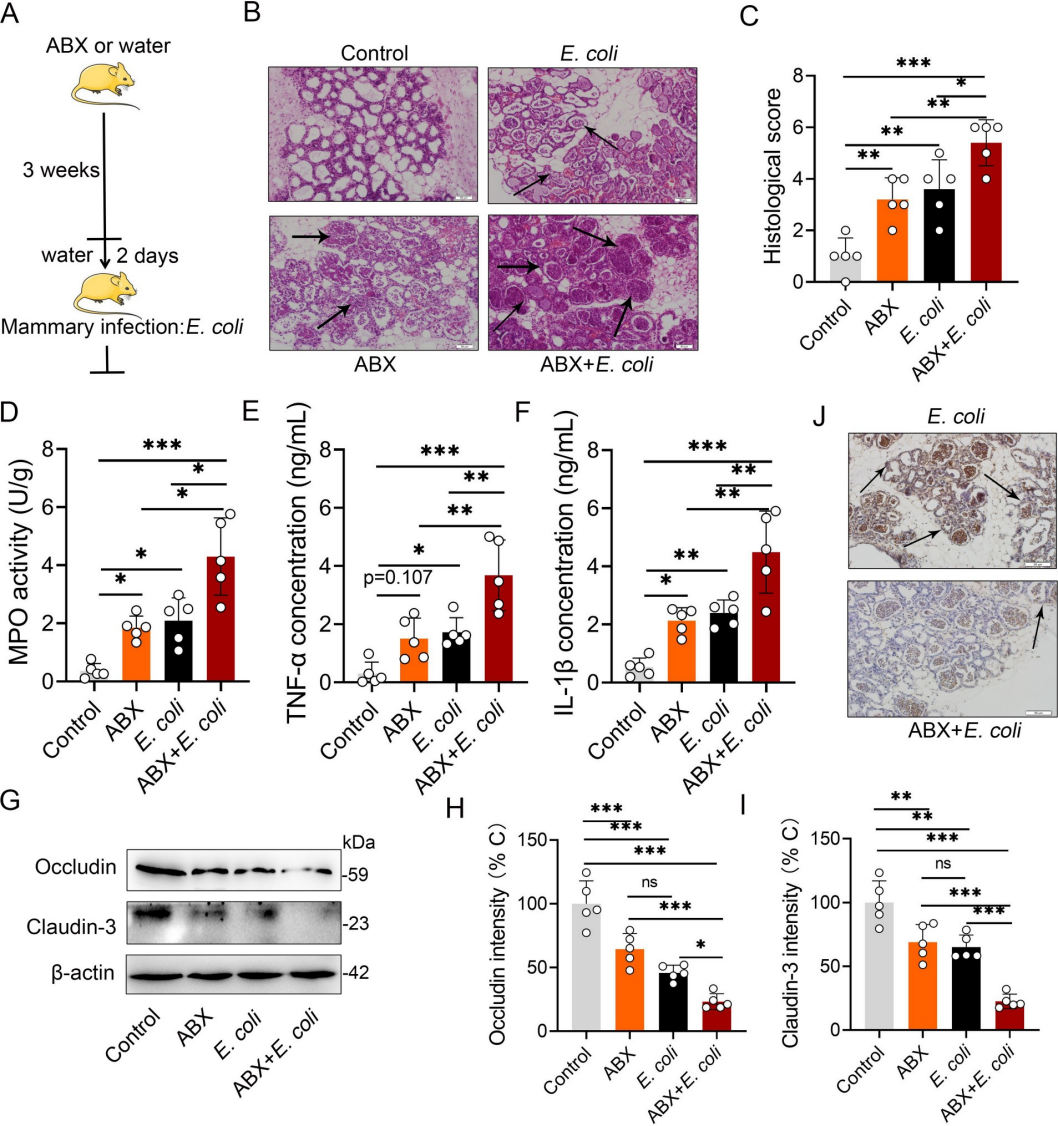
Gut dysbiosis aggravates *E*. *coli*-induced mastitis. **(A)** Schematic representation. The mice were treated with ABX (1 g/L ampicillin, neomycin sulfate and metronidazole and 0.5 g/L vancomycin) for 3 weeks, and ABX was removed for 2 days before mastitis was induced by *E*. *coli*. (**B)** Representative H&E staining of mammary gland tissue sections employed in histological analysis. The black arrow indicates inflammatory cells infiltration (scale bar, 50 μm). (**C)** Histological score according to H&E staining sections (n = 5). MPO activity (**D),** TNF-α (**E**) and IL-1β (**F**) were measured using ELISA on different groups mice (n = 5). Levels of occludin and claudin-3 protein (**G**) and intensity analysis (**H-I**) (n = 5). (**J)** Representative occludin antibody stained mammary gland sections (scale bar, 50 μm). The black arrow indicates positive staining. *p < 0.05, **p < 0.01 and ***p < 0.001 indicate significant differences from each group. ns, no significance. One-way analysis of variance was performed and the data are presented as the means ± SEM (C–F and H-I). ABX, a cocktail of antibiotics.

We then investigated whether impaired mammary AhR activation was associated with an increased inflammatory state in the mammary gland. We found that gut dysbiotic mice had obvious mastitis features, including increased acinus damage, histological score, MPO activity and pro-inflammatory tumor necrosis factor (TNF)-α and IL-1β expression **(Fig 4A–4F)** compared with the control mice. Increased inflammatory responses and acinus structure disruption commonly result in increased sensitivity to pathogen invasion [16]. We then tested whether impaired AhR activation contributes to *E*. *coli*-induced mastitis **(Fig 4A)**. As expected, upon *E*. *coli* stimulation, higher acinus damage and neutrophil infiltration **(Fig 4B and 4C)**, MPO activity **(Fig 4D)**, TNF-α and IL-1β expression **(Fig 4E and 4F)** were detected in the mammary glands of gut dysbiotic mice than in the control mice. We also showed that disrupting the intestinal microbiota impaired AhR activation, and deteriorated *E*. *coli*-induced barrier injury by reducing occludin and claudin-3 expression **(Fig 4G–4J).** Collectively, these results indicate that altering the intestinal microbiota could impair AhR activation and be associated with mastitis initiation and the development of *E*. *coli*-induced mastitis.

### Dietary tryptophan intervention improves *E*. *coli*-induced mastitis by activating AhR through microbiota metabolism

**Fig 5:**
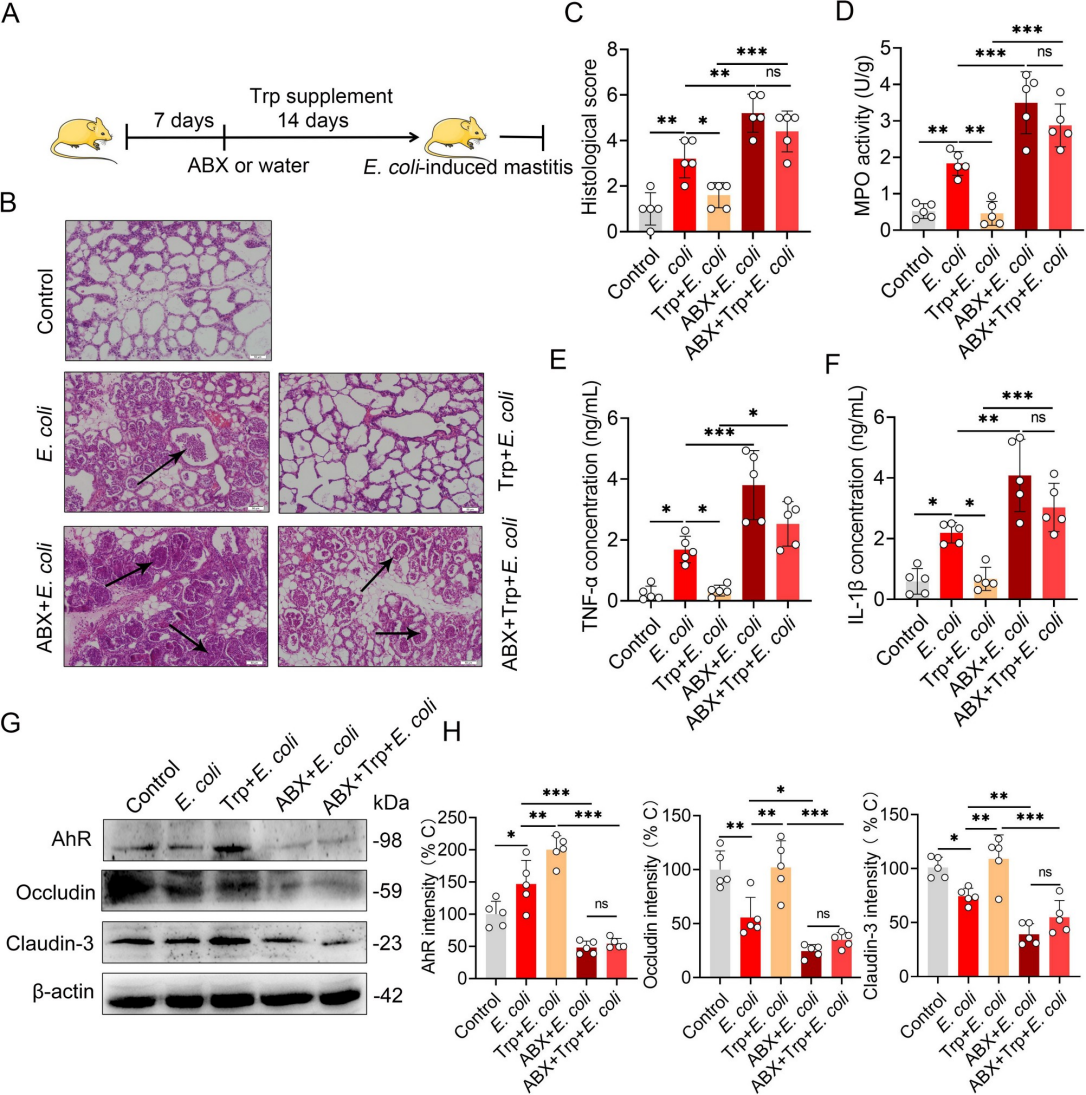
Tryptophan improves *E*. *coli*-induced mastitis by intestinal microbiota metabolism. **(A)** Schematic representation of tryptophan treatment. The mice were pretreated with ABX (1 g/L ampicillin, neomycin sulfate and metronidazole and 0.5 g/L vancomycin) or water for one week and their food was supplemented with or without tryptophan (1%) for two weeks, followed by modeling of the *E*. *coli*-induced mastitis (10^7^ CFU/50 μL by intra-breast injection). (**B**) Representative H&E staining of mammary gland tissue sections used in histological analysis. The black arrow indicates inflammatory cell infiltration (scale bar, 50 μm). (**C)** The histological score according to H&E staining sections (n = 5). (**D)** The MPO activity was assessed in mammary gland tissues from differently treated mice (n = 5). Inflammatory markers TNF-α (**E**) and IL-1β (**F**) were measured using ELISA on mice from different groups (n = 5). AhR, occludin and claudin-3 protein levels (**G**) and intensity analysis (n = 5) (**H**). ns, no significance, *p < 0.05, **p < 0.01, and ***p < 0.001 indicate statistical significance by one-way analysis of variance. The data are presented as the means ± SEM (C–F and H). ABX, a cocktail of antibiotics; Trp, tryptophan.

The intestinal microbiota could metabolize dietary tryptophan into AhR ligands and regulate AhR-associated host physiological function and disease development [13]. To investigate whether intestinal microbiota mediates the tryptophan metabolism, regulating AhR activation and mastitis pathogenesis, in addition to possibly improving mastitis outcomes by dietary consumption, we treated mice with tryptophan with or without ABX, followed by *E*. *coli* stimulation **(Fig 5A)**. We showed that, relative to *E*. *coli* stimulation, pretreating with tryptophan ameliorated *E*. *coli*-induced mastitis profiles by decreasing the histological score **(Fig 5B and 5C)** and several inflammatory markers **(Fig 5D–5F)**. In addition, supplementing with tryptophan also improved the blood-milk barrier integrity by increasing occludin and claudin-3 expression **(Figs 5G–5H and** S3**)**. However, the protective effects of tryptophan were abolished in the context of gut dysbiosis **(Figs 5A–5H and** S3**)**, leading to AhR activation by dietary tryptophan-derived ligand depending on the microbiota context [13,16,37].

**Fig 6:**
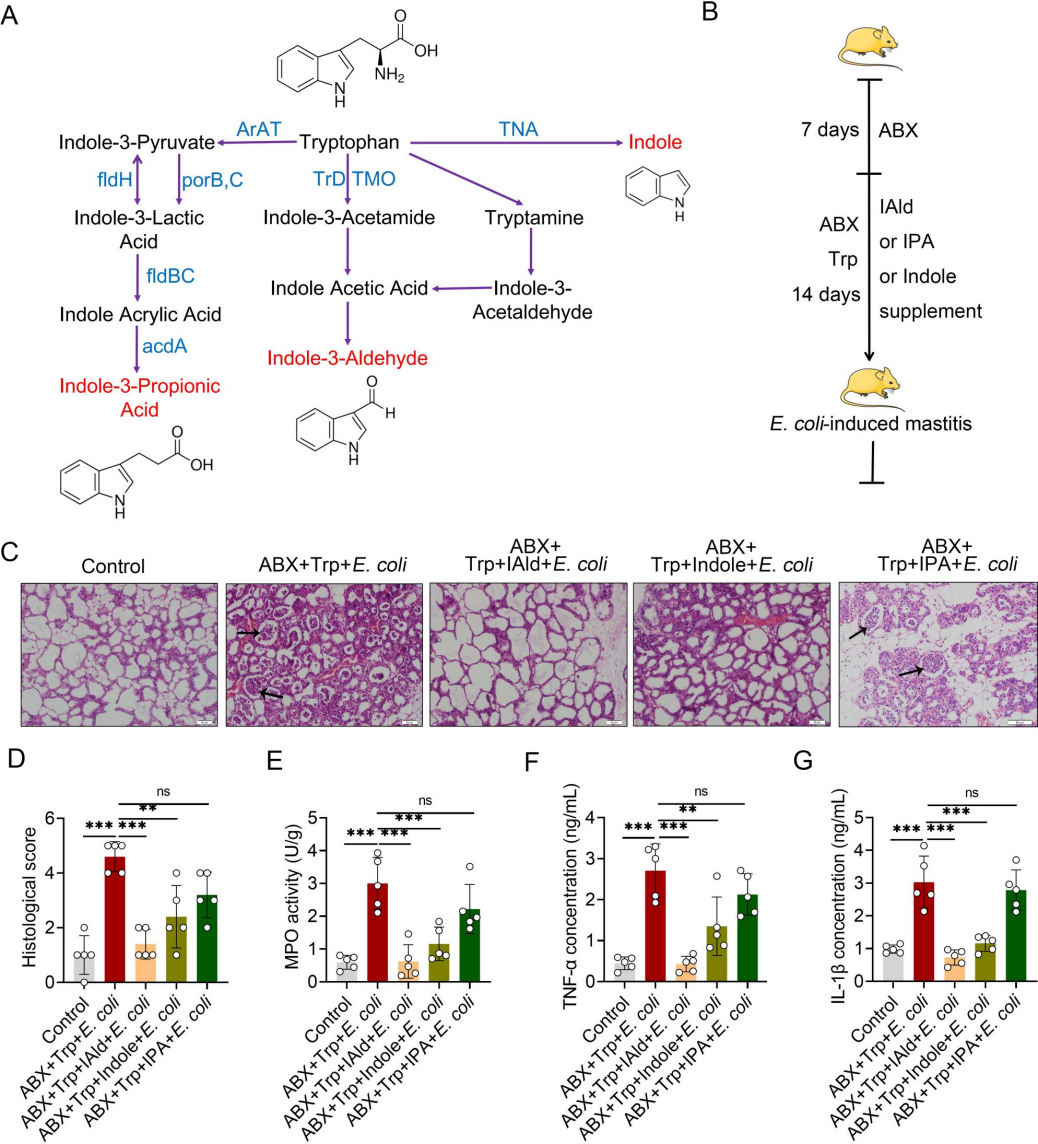
Tryptophan metabolized AhR ligands by intestinal microbiota ameliorate *E*. *coli*-induced mastitis in dysbiotic mice. **(A)** Tryptophan microbiota metabolism. (**B)** Schematic representation of AhR ligands rescue. The mice were treated with ABX for a week for microbiota depletion, followed by IAld, indole or IPA supplementation for 2 weeks in the context of tryptophan (1%) and ABX treatment before mastitis induced by *E*. *coli* stimulation (10^7^ CFU/50 μL by intra-breast injection). (**C)** Representative figures of H&E-stained mammary gland sections from tryptophan-derived AhR ligand supplemented mice. The black arrow indicates inflammatory cell infiltration (scale bar, 50 μm). **(D)** The histological score based on H&E-staining sections (n = 5). (**E)** The MPO activity from differently treated mice mammary glands (n = 5). Inflammatory cytokines TNF-α (**F**) and IL-1β (**G**) from different groups (n = 5). The data are presented as the means ± SEM (D-G). ns, no significance, *p < 0.05, **p < 0.05, and ***p < 0.001 indicate statistical significance by one-way analysis of variance. IAld, indole-3-aldehyde; IPA, indole-3-propionic acid; acdA, acyl-CoA dehydrogenase; AraT, aromatic amino acid aminotransferase; fldBC, phenyllactate dehydratase; fldH, phenyllactate dehydrogenase; porB, C, pyruvate: ferredoxin oxidoreductase B and C; TMO, tryptophan 2-monooxygenase; TNA, tryptophanase; TrD: tryptophan decarboxylase.

Tryptophan metabolism by the intestinal microbiota involves some small molecule production, including indole-3-aldehyde (IAld), indole and indole-3-propionic acid (IPA) in different catalysts [13] **(Fig 6A)**, which directly induces AhR activation. To confirm the effects of IAld, indole and IPA on *E*. *coli*-induced mastitis in the context of microbiota tryptophan metabolism, we supplemented ABX and tryptophan-treated mice with IAld, indole and IPA for 14 days by oral gavage **(Fig 6B)**. We demonstrated that compensation with IAld, indole but not IPA rescued mastitis features by reducing histological scores **(Fig 6C and 6D)** and inflammatory markers **(Fig 6E–6G)**. We also showed that IAld and indole but not IPA treatment increased tight junction expression (S4A–S4C Fig) but had no effects on *E*. *coli* growth (S5A–S5C Fig), indicating that induced AhR activation may account for inflammation limitations [16,18], Similar results have demonstrated that oral tryptophan-driven AhR ligands alleviated dermatitis [35] and EAE [21], suggesting that AhR activation by agonists from dietary sources may have systemic immunomodulatory effects. Overall, these results suggest the role of intestinal microbiota in the pathogenesis of mastitis through regulating AhR activation by metabolizing tryptophan into key active molecules.

### *Lactobacillus reuteri* (*L*. *reuteri*) with a strong ability to produce AhR ligands improves *E*. *coli*-induced mastitis in an AhR-dependent manner in mice

**Fig 7:**
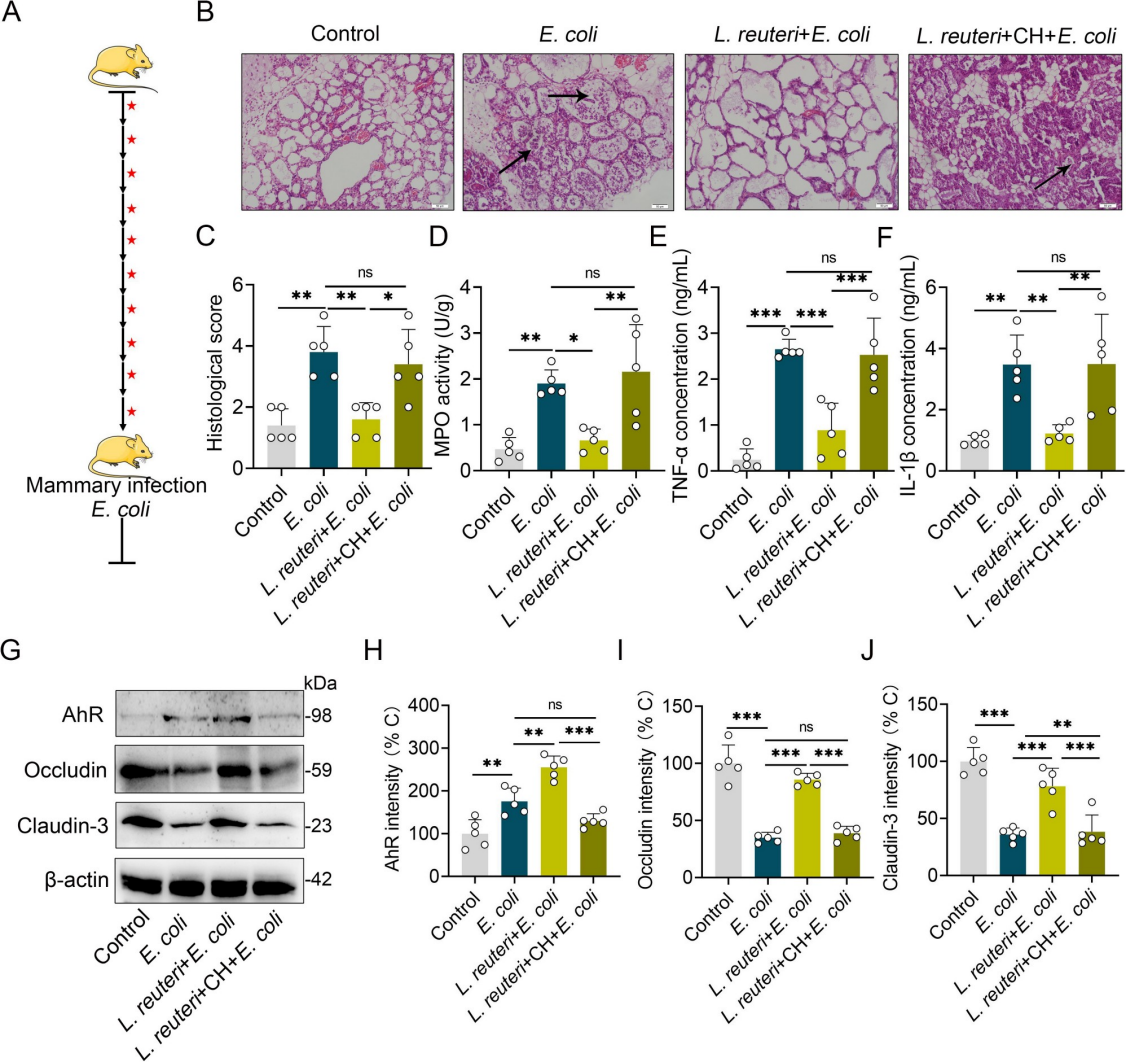
*Lactobacillus reuteri* (*L*. *reuteri*) ameliorates *E*. *coli*-induced mastitis by activating AhR. **(A)** Schematic representation of *L*. *reuteri* treatment. Black arrows indicate that the mice were given 10^9^ CFU/300 μL *L*. *reuteri* by oral gavage once every two days for 21 days. Red stars indicates CH223191 treatment (10 μg/mouse intraperitoneally) after each *L*. *reuteri* gavage. The mastitis model was induced by *E*. *coli* (10^7^ CFU/50 μL by intra-breast injection) at 21 days. **(B**) Representative H&E-staining of mammary gland tissue sections used in histological analysis. The black arrow indicates inflammatory cell infiltration (scale bar, 50 μm). (**C)** Histological score based on H&E stained sections (n = 5). (**D)** MPO activity in mammary gland tissues of different treated mice (n = 5). Inflammatory markers of TNF-α (**E**) and IL-1β (**F**) from different groups (n = 5). AhR, occludin and claudin-3 protein levels (**G**) and intensity analysis based on G (n = 5) (**H-J**). The data are presented as the means ± SEM (C–F and H-J). ns, no significance, *p < 0.05, **p < 0.05, and ***p < 0.001 indicate statistical significance by one-way analysis of variance. *L*. *reuteri*, *Lactobacillus reuteri*; CH, CH223191.

To explore the effects of specific probiotic interventions with high AhR ligand-producing capability on mastitis pathogenesis, we treated mice with different concentration of *L*. *reuteri* with the ability to produce AhR ligand for three weeks, followed by *E*. *coli* stimulation [19,20]. Our results demonstrated that *L*. *reuteri* consumption reduced *E*. *coli*-induced pathological damage to the mammary gland (S6A and S6B Fig), MPO activity (S6C Fig) and pro-inflammatory cytokines production (S6D and S6E Fig) in a dose-dependent manner. Similar to a previous study that *L*. *reuteri* intake ameliorated HFD-induced inflammatory responses and intestinal barrier dysfunction [20]. These results suggest that probiotic intake may yield fully beneficial physiological effects and is efficient at influencing the outcome of diseases. To verify the regulatory role of AhR in this process, we treated mice with an AhR antagonist after each *L*. *reuteri* oral gavage (**Fig 7A**) and found that AhR inhibition abolished *L*. *reuteri* mediated beneficial effects, including inflammation limitation and barrier repair (**Figs 7B–7J and** S7). Collectively, these results identify the beneficial role of AhR producer intervention on *E*. *coli*-induced mouse mastitis.

## Discussion

Mastitis is one of the most severe diseases for humans and animals, especially in the dairy industry [4,38]. Traditional views indicate that pathogen invasion pathogens into the mammary gland is the primary cause, while antibacterial and anti-inflammatory strategies that are locally administered by treating with antibiotics are inefficient for mastitis intervention [39]. Alterations in metabolites and intestinal microbiota have been described as being linked to mastitis [6,8,40], but their compositional and functional changes and potential roles in mastitis development are poorly understanding.

Microbiota-mediated tryptophan-based metabolites participate in IBD [19], metabolic disorders [20], dermatitis [35] and multiple sclerosis (MS) [21]. These effects are predominantly involved in AhR activation, which displays extensive physiological function through interactions with multiple transduction signaling pathways. Several studies have indicated a potential role for AhR in mastitis pathogenesis, and alterations in AhR ligand levels have been reported in mastitis [29,41]. In this study, we investigated whether impaired AhR activation by insufficient AhR ligand production though the disturbance of intestinal microbiota contributes to mastitis pathogenesis.

To investigate the protective effects of AhR activation in mastitis, we detected the AhR levels in the mammary glands of mice with mastitis caused by *E*. *coli*, which is one of the predominant pathogens involved in human and animal mastitis [4,5]. Mammary glands from mice with *E*. *coli-*induced mastitis have higher AhR expression, leading to the involvement of the AhR pathway in mastitis immunopathology [24,26]. Using an *E*. *coli-*induced mastitis model in mice, we pharmacologically activated the AhR pathway and showed that AhR activation alleviates *E*. *coli-*induced mastitis. Our results are consistent with previous studies showing that Ficz treatment alleviated colitis, EAE and celiac disease [19,21,36], leading to AhR activation regulating the host immunity in inflammatory and metabolic diseases.

The blood-milk barrier consists of intercellular tight junctions (TJs) and functions as gatekeepers for the preservation of nutrients and material exchanges between blood and milk [31]. In individuals with mastitis, the blood-milk barrier leaks, which are characterized by serum albumin translocation into milk and a reduced expression of TJs [31]. Here, we found that Ficz treatment ameliorates *E*. *coli*-induced damage to barrier function. Similar studies have indicated that Ficz treatment renews high fat diet (HFD) [20] or hypoxia-driven intestinal barrier disruption [42]. These studies implied the importance of the role of AhR in maintaining the barrier integrity [42,43]. The production of pro-inflammatory cytokines is attributed to mammary blood-milk barrier destruction, which is mediated by transcriptional signaling such as that of NF-κB [31,32]. Previous studies have shown that AhR activation transforms the pro-inflammatory program into an anti-inflammatory state through the regulation of cytokine production in both macrophages and dendritic cells (DCs) [16,17,30]. Indeed, we found that AhR activation inhibited the phosphorylation levels of NF-κB both in vivo and in vitro, leading to the AhR pathway control of inflammatory transcriptional signaling [33,44]. However, our results do not allow us to determine whether the restoration of the epithelial barrier integrity by AhR activation is a response to inflammation limitation in the context of mastitis. To confirm the role of AhR in the pathogenesis of *E*. *coli*-induced mastitis in mice, we treated mice with CH223191 and found that inhibiting of AhR activation aggravated *E*. *coli*-induced mastitis features, and reversed the beneficial role of Ficz, indicating that the AhR pathway is required for immune regulation in mastitis. The results of a previous study showed that FMT of GF mice using the feces of cows with mastitis could cause mastitis [6], which highlights the importance of the intestinal microbiota in mastitis pathogenesis. The intestinal microbiota accounts for AhR activation by producing AhR agonists from different sources [13]. To investigate the effects of the intestinal microbiota on AhR activation of the mammary gland and mastitis development, we treated mice with ABX to disrupt the intestinal microbiota [7]. Our study showed that intestinal dysbiosis impairs mammary gland AhR activation, initiates mice mastitis, as well as exacerbates *E*. *coli*-induced mastitis.

To confirm the role of intestinal microbiota in AhR activation and mastitis development, we treated mice with dietary tryptophan with or without gut dysbiosis. We concluded that dietary tryptophan supplementation improves *E*. *coli*-induced mastitis in a microbiota-dependent manner. Tryptophan from dietary sources is metabolized by the kynurenine pathway (KP) [45], the serotonin (5-hydroxytryptamine [5-HT]) production pathway [46] and the gut microbiota [13,18]. Intestinal microbiota-based tryptophan metabolism predominantly accounts for the production of AhR agonists, including IAld, indole and IPA, as documented by studies demonstrating that GF mice have abolished inductions of AhR ligands and low levels of AhR activity [19]. Kynurenine produced by host cells can also serve as an AhR agonist at high concentrations in vitro, while its low concentrations in different tissues are not sufficient to make it be a major AhR ligand in vivo [13,36]. Similar to ABX-treated mice, we showed impaired AhR activity in tryptophan-supplied dysbiotic mice. To confirm whether microbiota-metabolized AhR ligands mediate the protective effects of tryptophan in the mastitis context, we compensated tryptophan supplied dysbiotic mice with IAld, indole and IPA. We found that IAld and indole, but almost no IPA, rescued the tryptophan effects on *E*. *coli*-induced mastitis characterized by improved inflammatory responses and barrier integrity. Similar results have been found in EAE [21], indicating that different AhR ligand-binding capacities and downstream signaling activities may result in variant effects on disease outcomes [16,17,30].

*L*. *reuteri* has been shown to be engaged in the transformation of tryptophan into AhR ligands, especially IAld and indole [13,18]. Previous studies have shown that administrating *L*. *reuteri* ameliorates colitis, metabolic syndrome and celiac disease [19,20,36]. Indeed, treating mice with *L*. *reuteri* alleviates *E*. *coli*-induced inflammation and barrier injury, while inhibiting AhR activation reverses *L*. *reuteri’s* effects, suggesting that *L*. *reuteri* regulates mastitis development by producing AhR agonists [18,36]. *L*. *reuteri* can transform tryptophan into AhR agonists, but combination of *L*. *reuteri* and tryptophan did not further reduce disease severity in celiac disease mice [36], suggesting that a low abundance of *L*. *reuteri* is enough to produce sufficient AhR agonists and mediate disease outcomes [18,36].

Overall, we found that impaired AhR activity and ligand production by alterations in the intestinal microbiota exacerbated mastitis and that the mastitis score was ameliorated by AhR activation through compensation with AhR ligands or the correction of intestinal microbiota dysbiosis, suggesting that alterations in microbiota-dependent AhR signaling may facilitate the outcome of mastitis. Although our results did not allow us to identify whether microbiota-based AhR impairment is the primary factor or if it acts as a reinforcement event in the mastitis context, efficient protective effects through regulating of AhR signaling highlight the significant role of AhR in promoting the outcome of mastitis. Importantly, injury to tryptophan-based metabolites and AhR activation are also associated with mastitis in individuals [47,48]. In conclusion, our results provide evidence for microbiota-mediated metabolic disorders in the regulation of mastitis pathogenesis and suggest that targeting the intestinal microbiota to correct metabolic imbalance using dietary compensation is instrumental in the mastitis context, further serving as a basis for interventions in infectious and metabolic diseases based on seeking key metabolic molecules and improving of intestinal flora through supplementation with dietary components or probiotics.

## Material and methods

### Ethical statement

All animal experiments were subject to approval by the Institutional Animal Care and Use Committee (IACUC) of Jilin University (China). The full proposal was considered by the IACUC ethics committee, which approved the animal care and use permit license. All experiments comply with the manual of the care and use of laboratory animals published by the US National Institutes of Health.

### Materials

Tryptophan, indole, indole-3-propionic acid (IPA), indole-3-aldehyde (IAld), 6-formylindolo[3,2-b]carbazole (Ficz), 2-methyl-2H-pyrazole-3-carboxylic acid (CH223191) were purchased from Sigma Aldrich (St. Louis, MO, USA). The specific primary antibodies of AhR and Cyp1a1 were bought from Affinit Biosciences (OH, USA). Phosphorylation (p-) of p65 and IκB, p65, IκB and β-actin were bought from Cell Signaling Technology (CST, Boston, USA). Occludin and claudin-3 were bought from Bioss (Beijing, China). Enzyme-linked immunosorbent assay (ELISA) kit for TNF-α and IL-1β were bought from Biolegend (CA, USA). Myeloperoxidase (MPO) assay kit was obtained from Nanjing Jiancheng Bioengineering Institute (Nanjing, China).

### Animals

All specific pathogen free (SPF) grade Balb/c mice were purchased from the Experimental Animal Center of Baiqiuem Medical College, Jilin University (China). These Balb/c mice were mixed at a ratio of three females to one male in cages, and they were provided with enough water and breeding fodder until pregnancy. After pregnancy was confirmed, mice were subjected to different treatments.

### Eradication of the intestinal microbiota using an antibiotic cocktail

To disrupt the intestinal microbiota, the pregnant mice were treated with an antibiotic cocktail (ABX) as previously described [7]. In brief, 1 g/L metronidazole (Sigma), ampicillin (Sigma) and neomycin sulfate (Sigma), and 0.5 g/L vancomycin (Scientific Research Ievei) were added to drinking water for 3 consecutive weeks [7]. The antibiotics were removed from the drinking water for 2 days before establishing a model of mastitis using *E*. *coli* according to the following method [7].

### AhR agonist and antagonist treatments

To modulate AhR activation, CH223191 (10 μg/mouse) was intraperitoneally administered 1 h before 6-formylindolo (3, 2-b) carbazole (Ficz) application [35,49]. The Ficz was dissolved in dimethyl sulfoxide (DMSO) atconcentration of 1μg/μL, and 20 μL of Ficz solution was mixed with 280 μL of PBS [49]. Lastly, 1 μg/mouse of Ficz was administered via intraperitoneal injection 1 h before mouse mastitis model induction [49]. The control mice were treated with equal volumes of the vehicle.

### Dietary tryptophan supplementation and AhR ligand rescue experiments

To assess the effects of tryptophan on mastitis, the mice were treated with control diet (American Institute of Nutrition diet (AIN93G)) or control diet with 1% tryptophan for 2 weeks [23,50], with or without ABX (1 g/L metronidazole, ampicillin and neomycin sulfate, and 0.5 g/L vancomycin) pretreatment for one week and then they were treated throughout the experiment [21]. Composition of control diet was illustrated in S1 Table. For the AhR ligand rescue experiments, indole, indole-3-propionic acid (IPA), and indole-3-aldehyde (IAld) at 400 μg/20 g body weight were administered by oral gavage to mice for 14 consecutive days [21]. The control mice were treated by gavage with equal volumes of the vehicle (0.2% sodium carboxymethylcellulose and 0.25% polysorbate-80 in PBS) [50].

### AhR producing bacteria supplement experiment

In an experiment for evaluating the beneficial role of AhR ligand-producing *L*. *reuteri* on mastitis, the mice were given 10^7^, 10^8^ and 10^9^ CFU of *L*. *reuteri* CNCM I-5022 or vehicle (MRS broth supplemented with 0.05% L-cysteine and 15% glycerol) by oral gavage once every two days for 21 days [19,20,36], followed by mastitis model induction. To investigate the role of the AhR pathway in the beneficial effects of *L*. *reuteri*, the mice were treated with CH223191 (10 μg/mouse) intraperitoneally after *L*. *reuteri* (10^9^ CFU) oral gavage [19].

### Mouse mastitis model

The experimental mastitis model was induced according to previously described methods [7,32]. In brief, the mice were prepared, and their offspring were removed for 3 h before milk duct injection. Then the mice were anesthetized by using uratan (100 mg/kg) intraperitoneally and the fourth nipple was disinfected by using 75% alcohol. *E*. *coli* CVCC1418 (10^7^ CFU/50 μL) were injected through the milk duct at the fourth nipple of the mouse mammary gland by using a 100-μL syringes with a 30-gauge blunt needle. The control mice were anesthetized and injected with equal volume PBS. The mice were sacrificed 24 h after mastitis model establishment, and the mammary gland was collected aseptically and stored at -80°C for detection.

### Histology evaluation of mammary gland

All the mammary gland samples used for histological assessment were treated with 4% paraformaldehyde, embedded in paraffin and prepared as 5-μm paraffin sections (three sections per sample). The paraffin sections were stained with hematoxylin and eosin (H&E) and then detected using an optical microscope. The histological score of the mammary gland was determined according to the following scores [7]: (1) destruction of acinar structure: 0, no signs of destruction; 1, slight destruction; 2, moderate destruction; and 3, severe structure destruction; (2) inflammatory cell infiltration: 0, no cell infiltration; 1, slight; 2, moderate; and 3, severe.

### MPO activity determination

To test the degree of neutrophil infiltration, MPO activity was determined. 10% tissue homogenate was prepared and MPO activity was calculated according to the manufacturer’s certificate (Nanjing Jiancheng, China).

### Cytokines assays

To determine the pro-inflammatory cytokines expressions, 10% tissue homogenate from the mammary gland was prepared using PBS and Enzyme linked immunosorbent assay (ELISA) was performed according to the manufacturer’s instruction (Biolengend, USA).

### Real-time PCR

The total RNA from the tissues was extracted using TRIzol (Invitrogen, Carlsbad, CA, USA) and then treated successively with chloroform, isopropanol and 75% ethyl alcohol under RNase-free conditions. Quantitative RT–PCR was applied using TransStart Tip Green qPCR SuperMix (TransGen Biotech, Beijing, China) and then a FastStart Universal SYBR Green Master Mix (ROX) (Roche, Switzerland, Basel) in a Step One Plus apparatus (Applied Biosystems, Foster City, CA, USA). The reaction conditions were as follows: 52°C for 2 min, 95°C for 10 min, 95°C for 15 s and 60°C for 1 min for 45 cycles. The oligonucleotides used here are detailed in the S2 Table. To normalize the gene expression, GAPDH was used as an endogenous control and the 2^−ΔΔCt^ method was used. Specifically, the control group was used as a calibrator.

### Western blots analysis

Total protein samples were collected by using tissue protein extract (Thermo Fisher Scientific, USA), and the protein concentrations were measured using a BCA Protein Assay Kit (Thermo Fisher Scientific, USA). Target proteins were separated using 10% or 15% SDS-PAGE based on molecular size and then the proteins were bonded to 0.45 μm PVDF membranes following methanol treatment. After being blocked in 5% skim milk for 3 h at room temperature, the PVDF membranes were incubated with specific primary antibodies (1:1000 for AHR, Cyp1a1, p-p65, p-IκB, p65, IκB and 1:4000 for β-actin) at 4°C overnight. Furthermore, the PVDF membranes were incubated with Goat anti-rabbit IgG (1:20000) for 2 h at room temperature after being washed three times with TBST. Finally, the proteins were identified using the ECL plus western blotting Detection System.

### Immunohistochemical staining

Mammary gland paraffin sections were dewaxed as follows: xylene twice for 30min each, and 100%, 95% and 80% alcohol twice every 5 min. Then, the sections were subjected to for antigen retrieval using sodium citrate following phosphate buffer (PBS) wash. Prepared sections were treated with endogenous peroxidase blockers (SAP (Mouse/Rabbit) IHC Kit, MXB, China) for 40 min at room temperature followed by PBS washing 3 times per 5 min. The sections were then incubated with normal nonimmune goat serum (SAP (Mouse/Rabbit) IHC Kit, MXB, China) for 40 min at room temperature and then incubated with occludin antibody (1:200, diluted with 5% goat serum) overnight at 4°C. Furthermore, the sections were incubated with the secondary antibody (goat-anti rabbit IgG) for 30 min at room temperature after being washed with PBS3 times for 5 min each time. The sections were then incubated with horseradish peroxidase (HRP) (SAP (Mouse/Rabbit) IHC Kit, MXB, China) for 20 min at room temperature following PBS washing. After being washed 3 times for 5 min per wash with PBS, the sections were developed for 3 min using a color developing agent (SAP (Mouse/Rabbit) IHC Kit, MXB, China) under the microscope and terminated by water according to the color. The nuclei were staining with hematoxylin for 5 min followed by 1% muriatic acid alcohol differentiation and ammonium hydroxide treatment. Following dehydration, the sections were mounted utilizing neutral resins.

### Cell cultures and treatment

Primary mouse mammary epithelial cells (MMECs) were prepared as previously described [51] and cultured in Dulbecco’s modified Eagle’s medium (DMEM) supplemented with 10% fetal bovine serum (FBS) and 1% penicillin and streptomycin (Sigma Aldrich), at 37°C with 5% CO_2._ For the Ficz treatment test, MMECs (10^6^ cells/mL) were incubated in 6-well plates stimulated with Ficz (0.25, 0.5 and 1 μM) for 1 h followed by *E*. *coli* (10^7^ CFU) stimulation for 24 h and the cells were harvested by protein extract (Thermo Fisher Scientific, USA) for western blot analysis.

### Bacteria cultures

*L*. *reuteri* CNCM I-5022 was purchased from the Collection Nationale de Cultures de Microorganismes (CNCM) at the Institut Pasteur and grown in MRS (Haibo, Qingdao, China) broth with 0.05% L-cysteine at 37°C anaerobic conditions for 48 h. *E*. *coli* CVCC 1418 was cultured in lysogeny broth (LB, Haibo, Qingdao, China) at 37°C 180 r/min for 12 h to reach mid-log phase.

### Antibacterial test

In order to determine the effects of Ficz, IAld, indole and IPA on the growth of *E*. *coli*, Ficz (0.25, 0.5 and 1 μM), IAld (15, 30 and 60 μM), indole (0.25, 0.5 and 1 mM) and IPA (25, 50 and 100 μM) were added to the LB medium containing *E*. *coli* (10^7^ CFU/mL), and the growth status was evaluated by measuring optical density (OD) at 600 nm 24 h after incubation.

### Statistical analysis

For single comparisons, two-tailed Student’s t test was performed to calculate the p values. For multiple comparisons, one-way analysis of variance (ANOVA) was applied. *p* < 0.05 indicates statistical significance. GraphPad Prism 8 (San Diego, CA, USA) was used for the statistical analyses. The data are expressed as means ± SEM and representative data are one out of three independent experiments. The numerical data used in all figures are included in S1 Data.

## Supporting information

S1 Fig Effects of Ficz on NF-κB signaling pathway and *E. coli* growth in vitro. **(A)** Effects of Ficz on NF-κB activation assessed by western blotting. Mouse mammary epithelial cells (MMECs, 10^6^ cells/mL) were incubated in 6-well plate for 12 h. MMECs were pretreated with Ficz (0.25, 0.5 and 1 μM) for 1 h and stimulated with *E*. *coli* (multiplicity of infection is 10:1) for 24 h in the context of DMEM without ampicillin and streptomycin. The cells were harvested and the protein levels of NF-κB pathway were measured using western blotting (n = 3). (**B-C)** The relative intensity was analyzed (n = 3). The β-actin and control group were used as a calibrator. (**D)** Effects of Ficz on *E*. *coli* growth. *E*. *coli* was incubated in lysogeny broth (LB) with or without Ficz (0.25, 0.5 and 1 μM) for 24 h and the growth situation was assessed using spectrophotometer at 600 nm optical density (OD600). The data are presented as the means ± SEM (B–C). ns, no significance, ***p < 0.001 indicates statistical significance by one-way analysis of variance.
(TIF)

S2 Fig AhR is required for the pathogenesis of *E. coli*-induced mastitis. The mice were pretreated with or without CH223191 (10 μg/mouse intraperitoneally) for 1 h before Ficz administration (1 μg/mouse i.p.), followed by *E*. *coli*-induced mastitis (10^7^ CFU/50 μL by intra-breast injection). Twenty-four hours later, the mammary gland tissues were harvested and determined. (**A)** Representative images of H&E-stained mammary gland sections in different treated mice. The black arrow indicates inflammatory cell infiltration (scale bar, 50 μm). (**B)** Histological score based on H&E staining sections (n = 5). (**C)** The MPO activity of mammary gland tissues from differently treated mice (n = 5). TNF-α (**D**) and IL-1β (**E**) were assessed using ELISA from different groups (n = 5). AhR, occludin and claudin-3 protein levels (**F**) and intensity analysis (n = 5) (**G**). (**I)** Representative occludin antibody stained mammary gland sections (scale bar, 50 μm). Arrows indicate positive staining. The data are presented as the means ± SEM (B–E and G). ns, no significance, *p < 0.05, **p < 0.05, and ***p < 0.001 indicate statistical significance by one-way analysis of variance. CH, CH223191.
(TIF)

S3 Fig Tryptophan consumption increases occludin level in the mammary gland in the context of intestinal microbiota. A. The mice were pretreated with ABX (1 g/L ampicillin, neomycin sulfate and metronidazole and 0.5 g/L vancomycin) or water for 7 days and supplemented with or without tryptophan (1%) for two weeks, followed by *E*. *coli*-induced mastitis (10^7^ CFU/50 μL by intra-breast injection). The occludin expression was assessed using IHC by occludin antibody staining (scale bar, 50 μm). ABX, a cocktail of antibiotics; Trp, tryptophan.
(TIF)

S4 Fig Tryptophan metabolized AhR ligands by intestinal microbiota increased tight junction protein expression. The mice were treated with IAld, indole or IPA supplementation for 2 weeks in the context of tryptophan (1%) and ABX treatment followed by *E*. *coli* stimulation (10^7^ CFU/50 μL by intra-breast injection). (**A)** AhR, occludin and claudin-3 protein levels. (**B)** Intensity analysis based on A (n = 5). (C**)** Representative occludin antibody stained mammary gland sections (scale bar, 50 μm). Arrows indicate positive staining. The data are presented as the means ± SEM (B). ns, no significance, *p < 0.05, **p < 0.05, and ***p < 0.001 indicate statistical significance by one-way analysis of variance. IAld, indole-3-aldehyde; IPA, indole-3-propionic acid.
(TIF)

S5 Fig Effects of IAld, indole and IPA on *E. coli* growth. *E*. *coli* (10^7^ CFU) was incubated in lysogeny broth (LB) with IAld (**A**, 15, 30 and 60 μM), indole (**B**, 0.25, 0.5 and 1mM) or IPA (**C**, 25, 50 and 100 μM) for 24 h, respectively, and the growth situation was assessed using spectrophotometer at OD600.
(TIF)

S6 Fig *L. reuteri* alleviates *E. coli*-induced mastitis in a dose-dependent manner. The mice were treated with different concentration of *L*. *reuteri* (10^7^, 10^8^ and 10^9^ CFU) for 21 days. Then, the mice were treated with *E*. *coli* (10^7^ CFU/50 μL) by intra-breast injection. (**A**) Representative images of H&E-stained mammary gland sections in different treated mice. The black arrow indicates inflammatory cell infiltration (scale bar, 50 μm). (**B)** Histological score based on H&E staining sections (n = 5). (**C)** The MPO activity of mammary gland tissues from differently treated mice (n = 5). TNF-α (**D**) and IL-1β (**E**) were assessed using ELISA (n = 5). The data are presented as the means ± SEM (B–E). *p < 0.05, **p < 0.05, and ***p < 0.001 indicate statistical significance by one-way analysis of variance. ns, no significance,
(TIF)

S7 Fig *L. reuteri* increases occludin expression in the mammary gland. **(A)** The mice were given 10^9^ CFU/300 μL *L*. *reuteri* by oral gavage once every two days for 21 days with or without CH223191 treatment (10 μg/mouse intraperitoneally) after each *L*. *reuteri* gavage. Twenty-four hours after *E*. *coli* stimulation, the mammary gland tissues were harvested and occludin expression of mammary gland tissues were assessed using immunochemistry. Black arrows indicate occludin positive staining (scale bar, 50 μm).
(TIF)

S1 Table Ingredient and nutrient composition of the base diet (AIN93G). (DOCX)

S2 Table The oligonucleotides used in this study. (DOCX)

S1 Data Excel spreadsheet containing, in separate sheets, the data points presented in Figs 1–7 and S1–S7. (XLSX)
