# Supplementary material for: Aryl hydrocarbon receptor activation by Lactobacillus reuteri tryptophan metabolism alleviates Escherichia coli-induced mastitis in mice
Source: PLoS Pathog. 2021 Jul 23;17(7):e1009774. doi: 10.1371/journal.ppat.1009774 (PMC8336809; doi:10.1371/journal.ppat.1009774)
Supplement: S1 Table — (DOCX) [file ppat.1009774.s008.docx]

S1 table Ingredient and nutrient composition of the base diet (AIN93G).

| **Ingredient** |  |
| --- | --- |
| Carbohydrate (% of energy) | 63.9 |
| Protein (% of energy) | 18.8 |
| Fat (% of energy) | 17.2 |
| Energy (Kcal/g) | 3.8 |
|  |  |
| **Ingredient, g/kg** |  |
| Corn starch | 398 |
| Maltodextrin | 132 |
| Sucrose | 100 |
| Cellulose | 50 |
| Casein | 200 |
| L-cystine | 3 |
| Soybean oil | 70 |
|  |  |
| **Ingredient, g/kg** |  |
| Calcium | 5 |
| Phosphorus | 3 |
| Sodium | 1.02 |
| Potassium | 3.6 |
| Magnesium | 0.507 |
| Iron | 0.035 |
| Zinc | 0.03 |
| Copper | 0.006 |
| Selenium | 0.00015 |
|  |  |
| **Ingredient, unit/kg** |  |
| Thiamin (g) | 0.005 |
| Riboflavin (g) | 0.006 |
| Niacin (g) | 0.03 |
| Pyridoxine (g) | 0.006 |
| Folate (g) | 0.002 |
| Vitamin B_12_ (mg) | 0.025 |
| Vitamin A (IU) | 4000 |
| Vitamin D (IU) | 1000 |
| Vitamin E (IU) | 75 |
| Vitamin K (mg) | 0.75 |
| Choline (g) | 1.027 |
