# Supplementary material for: Aryl hydrocarbon receptor activation by Lactobacillus reuteri tryptophan metabolism alleviates Escherichia coli-induced mastitis in mice
Source: PLoS Pathog. 2021 Jul 23;17(7):e1009774. doi: 10.1371/journal.ppat.1009774 (PMC8336809; doi:10.1371/journal.ppat.1009774)
Supplement: S2 Table — (DOCX) [file ppat.1009774.s009.docx]

**S2 table** The oligonucleotides used in this study.

| Gene | Primer | Sequence(5′ to 3′) |
| --- | --- | --- |
| AhR | sense | 5′-GAGCACAAATCAGAGACTGG-3′ |
|  | antisense | 5′-TGGAGGAAGCATAGAAGACC-3′ |
| Il22 | sense | 5′-CATGCAGGAGGTGGTACCTT-3′ |
|  | antisense | 5′-CAGACGCAAGCA TTTCTCAG-3′ |
| Cyp1a1 | sense | 5′-CTCTTCCCTGGATGCCTTCAA-3′ |
|  | antisense | 5′-GGATGTGGCCCTTCTCAAATG-3′ |
| Cyp1b1 | sense | 5′-AATGAGGAGTTCGGGCGCACA-3′ |
|  | antisense | 5′-GGCGTGTGGAATGGTGACAGG-3′ |
| Reg3g | sense | 5′-TTCCTGTCCTCCATGATCAAAA-3′ |
|  | antisense | 5′-CATCCACCTCTGTTGGGTTCA-3′ |
| Reg3b | sense | 5′-ATGCTG CTCTCCTGCCTGATG-3′ |
|  | antisense | 5′-CTAATGCGTGCGGAGGGTATATTC-3 |
| GAPDH | sense | 5′-AACTTTGGCATTGTGGAAGG-3′ |
|  | antisense | 5′-ACACATTGGGGGTAGGAACA-3′ |
